# Injury and Poisoning Mortality Trends in Urban and Rural China from 2006 to 2020 Based on Age-Period-Cohort Analysis

**DOI:** 10.3390/ijerph19127172

**Published:** 2022-06-11

**Authors:** Xin Yuan, Changgui Kou, Min Zhang, Wenyuan Ma, Zhitao Tang, Haiyan Sun, Wenjun Li

**Affiliations:** 1 Department of Social Medicine and Health Management, School of Public Health, Jilin University, Changchun 130021, China; yuanxin20@mails.jlu.edu.cn (X.Y.); zhangm21@mails.jlu.edu.cn (M.Z.); wyma20@mails.jlu.edu.cn (W.M.); tangzt21@mails.jlu.edu.cn (Z.T.); sunhy19@mails.jlu.edu.cn (H.S.); 2 Department of Epidemiology and Biostatistics, School of Public Health, Jilin University, Changchun 130021, China; koucg@jlu.edu.cn

**Keywords:** injury and poisoning, mortality, urban and rural, China, Joinpoint regression, age–period–cohort model

## Abstract

Injury and poisoning, common public health problems, currently rank fifth among the causes of death in China. In this study, we aimed to analyze the trends and influencing factors of injury and poisoning mortality in urban and rural China using an age-period-cohort model. Crude mortality data for injury and poisoning by sex, age group, and region were obtained from the China Health Statistical Yearbook (2006–2020). Age-standardized mortality rates for injury and poisoning in urban and rural areas were estimated using the Seventh Census of China 2020 population. The trends of injury and poisoning mortality were assessed using Joinpoint analysis. Age–period–cohort models were used to explore the age, period, and birth cohort effects affecting mortality risk. Over a 15-year period, age-standardized mortality rates decreased from 28.81/100,000 in 2006 to 24.78/100,000 in 2020 in urban areas and from 45.49/100,000 to 44.39/100,000 in rural areas. In the male population, the annual change in mortality was −0.4% (95% CI = −1.8%, 1.0%) in urban areas and −1.0% (95% CI = −1.9%, 0.0%) in rural areas. In the female population, the annual change in mortality was −1.2% (95% CI = −2.3%, −0.1%) in urban areas compared with −1.6% (95% CI = −3.1%, −0.1%) in rural areas. The age–period–cohort model showed a significant increase in urban and rural mortality rates starting at ages 49 and 39 years. Both showed a decline followed by an increase in the period. The cohort from 1929 to 2013 showed an overall trend of increasing and then decreasing. From 2006 to 2020, the overall injury and poisoning mortality rates in China showed a decreasing trend, and the mortality rates decreased faster in women than in men and in rural areas than in urban areas. Age effects were the most important risk factors for changes in injury and poisoning mortality. The results of this study will help researchers explore the possible causes of mortality changes in urban and rural areas and provide a scientific basis for injury and poisoning prevention and control priorities and policy formulation in China.

## 1. Introduction

As a major public health problem that poses a serious threat to human health and life, injury and poisoning have a worldwide impact that cannot be ignored [1]. According to the World Health Organization, more than 5 million people die each year from injuries and poisoning, accounting for 9% of all deaths worldwide, and it is predicted that road traffic injuries and falls will rise to the 7th and 17th causes of death worldwide, respectively, from 2012 to 2030 [2]. In China, injuries and poisonings account for approximately 800,000 deaths per year, or 7.45% of total population deaths, and they are the number one cause of death for people under 45 years of age [3,4]. The top three causes of death are road traffic injuries, falls, and suicide.

In fact, injuries and poisoning, because they are common and frequent and have high mortality and disability rates, not only increase the economic burden on the national health system but also indirectly affect the level of productivity and social development. Previous studies have shown that productivity losses due to injuries and poisoning account for 21–81% of the economic burden of disease [5]. In China, the most populous developing country in the world with a population of 1.3 billion and rapid economic development, risk factors such as traffic congestion and work stress are becoming increasingly serious [6]. They generate direct medical costs and indirect economic losses of up to CNY 70 billion because of expensive medical care and loss of productivity, posing a public health threat to economic development [7]. This further indicates that injury and poisoning have an impact on national policies, health care resource allocation, and economic development.

Although our country currently shows a decreasing trend in injury and poisoning, there are still sex and urban–rural differences. Globally, Serbia and Canada have explored the impact of aging and mental health services on disease mortality by gender subgroups, respectively [8,9]. The United States, Lithuania, Poland, and many other countries have extensively conducted epidemiological analyses of related disorders from an urban–rural perspective [10,11,12,13]. However, few epidemiological descriptions of injury and poisoning from an urban–rural perspective are available for mainland China. Only a few studies have reported on gastric cancer, diabetes mellitus, and cardiovascular diseases from an urban–rural perspective [14,15,16]. Therefore, it is necessary for us to study these issues from this perspective.

Existing studies have shown that government policies can effectively prevent or reduce the occurrence of injury and poisoning. For example, in the United States, 20–30% of respondents increased their support for legislation through the implementation of relevant policies and regulations, and prevention workers can use this support to legislate measures to reduce mortality [17,18]. In China, program interventions and health coaching have been implemented through the new health care system reform and the “Health China 2030” plan [2].

In previous studies, mortality data from a region, hospital, or city were analyzed. Alternatively, a subcategory, such as drowning or accidental poisoning, has been selected under this broad category for analysis [19,20]. These issues are less frequently studied under the broad category of injury and poisoning, and therefore, this paper allows for not only an understanding of recent trends in injury and poisoning mortality in urban and rural areas but also an analysis of important nodes of mortality change. Multisector cooperation with the public health sector is an important way to prevent and control the occurrence of injury and poisoning. Effective prevention and control of injury and poisoning will help increase the life expectancy of our population, improve its health status, and reduce the burden on society [2].

## 2. Materials and Methods

### 2.1. Data Sources

Crude mortality data for injury and poisoning by age group and region were obtained from the China Health Statistical Yearbook (2006–2020) [21], a continuous, nationally representative mortality surveillance program for the Chinese population that is publicly released by the National Health and Family Planning Commission. In 2000, the system was expanded from the 13 cities covered in the 1950s to 36 cities and 90 counties, covering approximately 110 million people. In 2012, the system was expanded to 319 monitoring sites (138 counties and 181 districts), covering approximately 230 million people [22].

Injury and poisoning mortality rates for urban and rural residents were selected from the “causes of death from disease or injury” section of the yearbook, but injury and poisoning mortality rates for towns and villages were not reported separately in the yearbook, so previous studies were used for reference [23]. The annual population of towns and villages came from the China Demographic Statistical Yearbook (1991–2006) and the China Population and Employment Statistical Yearbook (2007–2020). The data years were from 2006 to 2020 (fifteen years).

### 2.2. Statistical Analysis

Coarsening rates and age-standardized mortality rates were calculated. This study focused on 5–79-year-old (5-, 10-, 15- …… 70-, 75-) populations, in which truncation was chosen because of missing data on injuries and poisonings in China for those under 5 and over 80 years of age. We used the Seventh Census of China 2020 population stratified by age, sex, and region to calculate age-standardized mortality rates for men and women in urban and rural areas.

#### 2.2.1. Joinpoint Regression Analysis

We used the Joinpoint regression program software developed by the National Cancer Institute to create Joinpoint regression analyses to estimate the annual percentage change (APC) for each segment and the average annual percentage change (AAPC) for the entire period, which consists of several consecutive linear segments that are commonly used to describe trends in outcomes, also known as segmented regression, fold regression, and multistage regression [24]. Each spline indicated a significant change in the trend of the line segment separated by that spline, and the corresponding 95% confidence interval (CI) was calculated and tested for significance using the weight–BIC method to determine whether the AAPC was significantly different between 0 and α = 0.05.

#### 2.2.2. Age–Period–Cohort Model

Age–period–cohort models can estimate the risk of disease morbidity or mortality in a given population, adjusting for age, period, and cohort and can reflect the trends in disease across ages, periods, and cohorts [25]. The model is based on a Poisson log-linear model, which is widely used to avoid linear dependence issues (period = age + cohort) and to separate the three effects of age, period, and cohort [26].

In this study, the three parameter effects in the age–period–cohort model setup were fitted separately using the three nodes of age, period, and cohort variables to model mortality data by sex and urban or rural area. Lower values of the AIC and BIC criteria indicated a better model fit, and the age–period–cohort model was performed using the Stata16 APCfit [27].

## 3. Results

Overall, there was a decreasing trend in injury and poisoning mortality in China. Age-standardized mortality rates decreased from 28.81/100,000 in 2006 to 24.78/100,000 in 2020 in urban areas and from 45.49/100,000 to 44.39/100,000 in rural areas. We analyzed the changes in crude mortality rates and age-standardized mortality rates for the four subgroups by Joinpoint regression, as shown in Table 1 and Table 2, respectively. In terms of crude mortality, all four subgroups showed an overall decreasing trend in mortality from 2006 to 2020. Mortality rates for urban men, rural men, and rural women first increased and then decreased, with the turning points occurring in 2016, 2012, and 2008, respectively. The mortality rates for rural women declined the fastest (AAPC = 0.9%; 95% CI = −2.6–0.8%), and those for rural men declined the slowest (AAPC = 0.6%; 95% CI: −1.7–0.6%). The age-standardized mortality rates were 0.4% (CI = −1.8–1.0%) and 1.2% (CI = −2.3–0.1%) for urban men and urban women, respectively. Rural men and rural women showed a trend of increasing and then decreasing mortality rates, with the turning points occurring in 2012 and 2008, respectively. The rate of decline was slower for rural men (AAPC = 1.0%; 95% CI = −1.9–0.0%) than for rural women (AAPC = 1.6%; 95% CI = −3.1–0.1%).

In analyzing the trends of age-specific mortality rates for urban age groups, we found that the overall trend of change for urban men was first a decline and then a rise with age, with an inflection point at age 55 and some fluctuations in the rate of change. The rate of decline for urban men slowed from 3.3% to 0.9% for each age group in the age range of 5 to 20 years, and in the age range of 20 to 55 years, the rate of decline increased from 1.3% to a peak of 5.4% and then slowed to 1.4% for each age group. From 55 to 79 years of age, the rate of increase in mortality rose with increasing age, rising from 1.0% to 2.4% and then slowing to 0.8%. Urban women maintained an overall decline in mortality rate in the 5- to 65-year age range, with a rebound in the 10- to 15-year age group and an increase in mortality (AAPC = 2.5%; 95% CI = 0.4–4.7%). The fastest decline was seen in the 40-year age group (AAPC = 6.3%; 95% CI = −10.1–2.4%). For the age group of 65 to 70 years, a small increase occurred, with no more than a 1.0% rise (see Table A1).

Analysis of the mortality trends by age group in rural areas showed that the mortality rate of rural men first declined from ages 5 to 25 years, increased slightly from age 25 to 30 years (AAPC = 0.3%; 95% CI = −2.4–3.0%), and declined overall from age 30 to 60 years. The fastest decline was seen in the 40-year-old age group (AAPC = 5.4%; 95% CI = −8.2–2.6%). Starting at age 60, mortality increased with age. Rural women maintained a general decline in mortality at all ages from 5 to 79 years, with mortality rates increasing for ages 10 to 15 years and 75 to 79 years (AAPC = 1.9%; 95% CI = −5.0–9.3% and AAPC = 0.3%; 95% CI = −1.1–1.7%, respectively). Meanwhile, the fastest decrease in mortality was observed from ages 40 to 45 years (AAPC = 7.4%; 95% CI = −9.4–5.3%) (see Table A2).

For injury and poisoning mortality change trends in China, it was equally important to explore changes across age groups, observation periods, and birth cohorts. To facilitate the unfolding of age, period, and cohort trends in injury and poisoning mortality in China, the age, period, and cohort were segmented according to the actual situation. The ages were divided into 15 age groups with 5 years per age group: 5–9 years, 10–14 years …, and 75–79 years. The relationship between age, period, and cohort was as follows: birth cohort = period-age, with a total of 17 birth cohorts in the observed population: 1929–1933 … 2009–2013.

**Figure 1:**
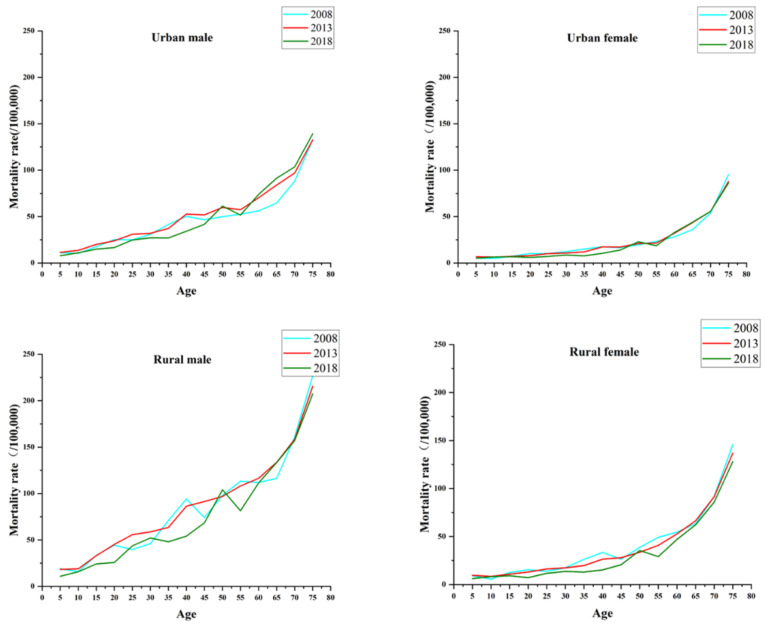
The trends of injury and poisoning mortality in China by gender and region with age.

The trends for injury and poisoning mortality in China by gender and region with age are shown in Figure 1. As a whole, the mortality rates increased with age throughout the period observed. The increase was slower and less volatile in urban areas than in rural areas and in women than in men. The mortality rate for rural men was much greater than that for the other three subgroups from ages 55 to 59 years, and it increased the most with age. The age effect showed an inflection point in the age group of 45–49 years in urban areas. In other words, the effect coefficients for urban men and urban women decreased with age from 5 to 49 years and increased with age after 49 years, with effect coefficients at the inflection points of −0.994 and −1.009 for men and women, respectively. There was a similar age effect in rural China, and the lowest age effects for rural men and rural women occurred at the age of 35–39 years, with effect coefficients of −0.518 and −0.525 (see Table A3 and Table A4).

**Figure 2:**
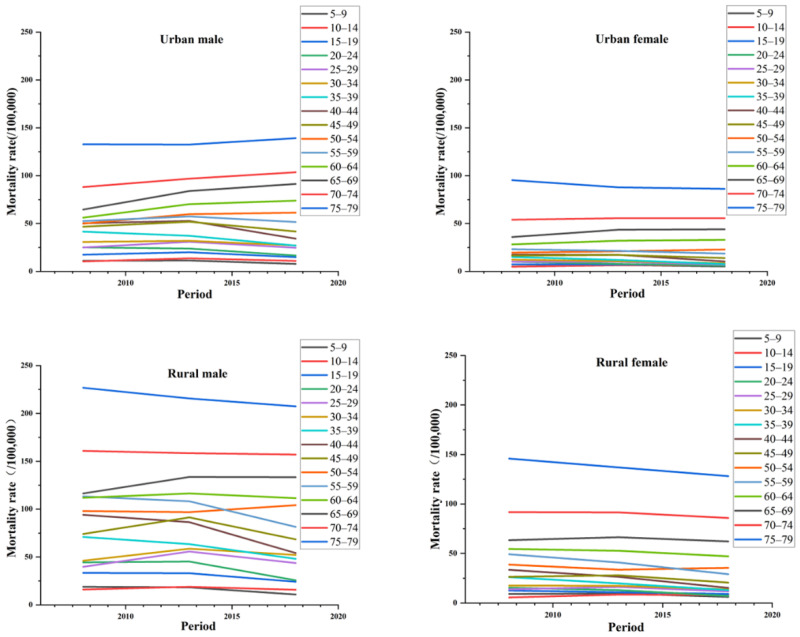
The trends of injury and poisoning mortality in China by gender and region with period.

In terms of the period effect, the trends of injury and poisoning mortality in China by gender and region are shown in Figure 2. The figure shows that the age group of 60–79 years showed a decreasing trend relative to the other three subgroups, except for urban men. The change was more obvious for urban men starting from age 40, and it showed a more pronounced increase with age starting at age 60 for urban women. It showed more significant fluctuation from 25 to 60 years of age in rural men. In terms of period effects, the overall risk of death for the four subgroups of injury and poisoning in China increased as time progressed during the observation period, with all four subgroups showing a decreasing and then increasing trend (see Table A3 and Table A4). From the perspective of clinical medicine, the continuously increasing period effect may be associated with the imperfection of medical treatment and the aging of the population, thus implying that the time factor is also an important factor in the increasing mortality from injury and poisoning in China.

**Figure 3:**
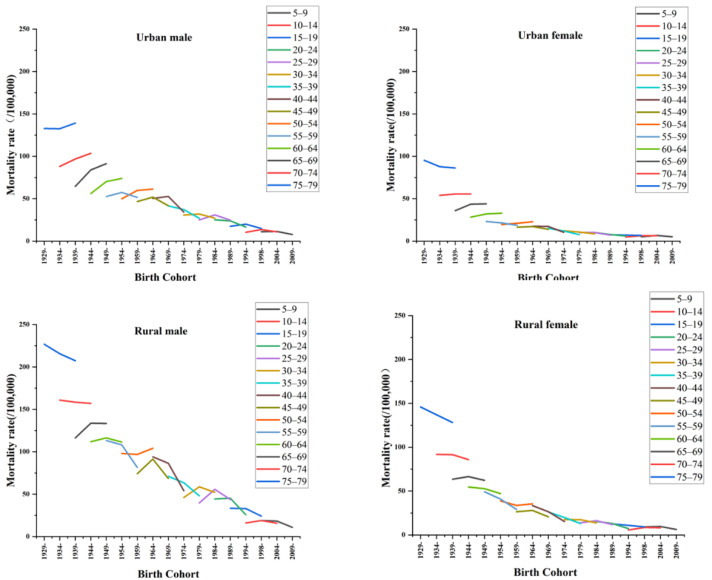
The trends of injury and poisoning mortality in China by gender and region with birth cohorts. Note: Birth cohort = period-age, the earlier the cohort (1929–1933) indicates that the current population is elderly, and the later the cohort (2009–2013) indicates that the current population is adolescent. Each colored line segment represents 15-year mortality trends for different birth cohorts.

The trends of injury and poisoning mortality in China by gender and region with birth cohorts are shown in Figure 3. In general, younger cohorts had a lower risk of injury and poisoning death than older cohorts. From age 5 to 39 years, all four subgroups showed an increasing and then decreasing trend. From age 40 to 44 years, it showed a gradually decreasing trend among rural women, and in the remaining three subgroups, it showed an increasing and then decreasing trend. With regard to cohort effects, people born from 1959 to 1963 had the highest cohort effects for injury and poisoning mortality, and the risk of death was 1.146, 1.190, 0.651, and 0.689 for urban men, urban women, rural men, and rural women, respectively (see Table A3 and Table A4).

## 4. Discussion

Studies of injury and poisoning from an urban–rural perspective are uncommon in China. In this study, we first used Joinpoint regression analysis to find an overall decreasing trend with some fluctuation in mortality across subgroups, after initially eliminating the effect of demographic composition on injury and poisoning mortality. The decline was slower in men than in women, which may be due to the differences in their personalities, lifestyles, and psychological stress. Because of the differences in the social division of labor, work pressure, and family roles, men tend to be more vulnerable to stress than women and have higher exposure to risk factors related to injury and poisoning death than women. Moreover, we found a higher mortality rate in men than in women, which is consistent with reports from many parts of China [28,29,30]. Therefore, it is necessary to improve safety awareness education and popularization among men and pay attention to men’s social and psychological health in response to these factors [31,32,33].

Second, we found a slower decline in urban areas than in rural areas, which might be due to differences between urban and rural areas in terms of medical resources, economic level, health environment, and education level. The investment and increase in funding for primary health service programs in China, the upgrading of equipment in medical institutions, and the improvement of the access environment have produced a virtuous cycle that has also contributed to the reduction in injury and poisoning mortality in rural areas [34]. At present, the prevention and care of injury and poisoning in rural China have achieved initially good results, and prevention and treatment under the leadership of the government need to be further improved and adjusted between urban and rural areas [35].

Regarding the changes in injury and poisoning mortality trends by age in urban and rural China during the observation period, we found that the rate of death first decreased and then increased in the four subgroups, with the inflection point mostly existing at the node of 55 years of age, indicating that with the increase in age, the risk of death increased; as the physical function of the elderly decreases, their reactions become slower, and chronic diseases and complications appear [36,37]. This suggests that the elderly population needs to be given adequate attention and should be focused on as a high-risk group for injury and poisoning prevention.

By analyzing age, period, and cohort effects through the age–period–cohort model, we found that age was the most important risk factor for injury and poisoning in China for the following two possible reasons. (1) As people age, older people have worse physical health, which can easily lead to the emergence of underlying diseases and complications. For example, older people are more likely to fall and have serious complications. (2) The overall aging of the population is increasing the number of older people, and along with the emergence of mental health problems, there is a higher risk of suicidal tendencies [38,39].

The period effects of the risk of death from injury and poisoning for urban and rural residents in China from 2006 to 2020 changed little and remained in the range of 0.5. As time progressed, mortality showed a decreasing and then increasing trend in the four subgroups. This indicates that the risk of death has increased in recent years. We need to pay more attention, establish a relevant disease burden assessment system, and call for the participation of the whole society in prevention under the guidance of the government.

The cohort effect of injury and poisoning deaths in rural and urban China showed an increasing and then decreasing trend. This may be related to the new health care system reform carried out in 2009. Under the new health care system reform, to balance the allocation of health resources in urban and rural areas, the government and related departments can effectively increase financial support according to the actual gap while clearly defining their own responsibilities and missions [40]. Not only can the relevant policies attract high-quality health talent for grassroots employment, but they can also strengthen the flow of talent between urban and rural areas by establishing special allowances for grassroots health personnel, optimizing the route of talent selection and training and improving the talent recruitment system [41]. At the same time, through the construction of medical associations and medical communities, the organizational structure and service content of hospitals and grassroots medical institutions can be effectively integrated to promote the creation of high-quality resources and provide convenient channels for grassroots medical personnel to improve their business capabilities [42].

In general, injury and poisoning mortality rates in urban and rural China show a declining trend, which is closely related to the continuous improvement of infrastructure in recent years, the soundness of the medical system, the improvement of medical standards, and the concerted efforts of government agencies in multiple sectors in China. Therefore, many experts and scholars have also put forward many prospective suggestions [43] through active and passive interventions to raise people’s safety awareness using publicity and education on the one hand, and to change personal perceptions and behavioral habits starting from themselves on the other hand. These suggestions also focus on vulnerable groups and the role of the community in prevention and control [44].

## 5. Conclusions

Injury and poisoning mortality rates in China can be described as higher for men than for women and higher in rural areas than in urban areas. There is currently an overall decreasing trend, and the rate of decline is faster for women than for men and in rural areas than in urban areas. A strong age effect for mortality, as well as smaller period and cohort effects, was found using the model. The mortality rate increased with age, and in terms of mortality levels, they were higher for men than for women and higher in rural areas than in urban areas, so men, older populations, and rural areas deserve more attention as potential high-risk populations and high-risk areas. Through national policy implementation, multisector coordination and cooperation, and health knowledge dissemination, we can reduce the urban–rural gap and improve the overall health of the population.

## Figures and Tables

**Table 1:** Changes in crude mortality rates of injury and poisoning by gender and region.

		Urban		Rural	
		Male	Female	Male	Female
segment	range	2006–2016	2006–2020	2006–2012	2006–2008
	APC (%)	1.7	−0.7	2.5	5.8
	APC 95% CI (%)	(−0.0, 3.4)	(−1.7, 0.2)	(0.2, 4.8)	(−6.7, 19.9)
	t	2.2	−1.7	2.5	1.0
	*p*	0.0052	0.119	0.034	0.341
segment	range	2016–2020		2012–2020	2008–2020
	APC (%)	−6.3		−2.8	−2.0
	APC 95% CI (%)	(−11.8, −0.4)		(−4.3, −1.3)	(−2.8, −1.2)
	t	−2.4		−4.2	−5.6
	*p*	0.038		0.002	<0.001
AAPC (%)		−0.7	−0.7	−0.6	−0.9
AAPC 95% CI (%)		(−2.5, 1.2)	(−1.7, 0.2)	(−1.7, 0.6)	(−2.6, 0.8)
Z		−0.7	−1.7	−1.0	−1.1
*P*		<0.1	<0.1	<0.1	<0.1

**Table 2:** Changes in standardized mortality rates of injury and poisoning by gender and region.

		Urban		Rural	
		Male	Female	Male	Female
segment	range	2006–2020	2006–2020	2006–2012	2006–2008
	APC (%)	−0.4	−1.2	2.4	6.8
	APC 95% CI (%)	(−1.8, 1.0)	(−2.3, −0.1)	(0.3, 4.4)	(−4.8, 19.9)
	t	−0.7	−2.3	2.6	1.3
	*p*	0.502	0.042	0.026	0.230
segment	range			2012–2020	2008–2020
	APC (%)			−3.4	−2.9
	APC 95% CI (%)			(−4.6, −2.1)	(−3.6, −2.2)
	t			−5.8	−9.3
	*p*			<0.001	<0.001
AAPC (%)		−0.4	−1.2	−1.0	−1.6
AAPC 95% CI (%)		(−1.8, 1.0)	(−2.3, −0.1)	(−1.9, 0.0)	(−3.1, −0.1)
Z		−0.7	−2.3	−1.9	−2.0
*P*		<0.1	<0.1	<0.1	<0.1

## Data Availability

The data in this study were obtained from the China Health Statistical Yearbook and the National Bureau of Statistics. The data are publicly available.

## Appendix A

**Table A1:** The temporal trends in age-specific mortality rates for each age group in urban regions using Joinpoint regression analysis.

	Male				Female			
Age	Range	APC (%, 95% CI)	*p*	AAPC (%, 95% CI)	Range	APC (%, 95% CI)	*p*	AAPC (%, 95% CI)
5-	2006–2014	1.0 (−3.0, 5.1)	0.601	−3.3 (−6.2, −0.4)	2006–2008	−23.4 (−49.8, 16.9)	0.179	−2.3 (−9.0, 4.9)
	2014–2020	−8.8 (−14.1, −3.2)	0.007		2008–2012	18.7 (−2.9, 45.1)	0.083	
					2012–2020	−5.9 (−9.0, −2.6)	0.004	
10-	2006–2010	−2.8 (−12.8, 8.4)	0.559	−0.7 (−6.8, 5.8)	2006–2020	2.5 (0.4, 4.7)	0.025	2.5 (0.4, 4.7)
	2010–2013	16.7 (−14.8, 59.9)	0.284					
	2013–2020	−6.2 (−9.7, −2.6)	0.005					
15-	2006–2013	4.4 (−1.5, 10.7)	0.132	−0.9 (−4.4, 2.7)	2006–2020	−0.1 (−2.1, 2.0)	0.955	−0.1 (−2.1, 2.0)
	2013–2020	−6.0 (−11.2, −0.6)	0.034					
20-	2006–2013	1.6 (−3.2, 6.6)	0.459	−1.3 (−7.1, 4.9)	2006–2010	7.3 (−7.9, 25.1)	0.310	−0.5 (−7.3, 6.8)
	2013–2017	−15.0 (−29.1, 1.9)	0.072		2010–2017	−11.7 (−18.6, −4.1)	0.009	
	2017–2020	12.6 (−9.0, 39.3)	0.229		2017–2020	18.7 (−10.9, 58.0)	0.201	
25-	2006–2011	0.6 (−6.7, 8.6)	0.848	−2.4 (−6.5, 1.8)	2006–2015	0.0 (−3.3, 3.4)	0.991	−3.8 (−6.8, −0.7)
	2011–2015	9.7 (−3.8, 25.2)	0.141		2015–2020	−10.3 (−17.4, −2.6)	0.015	
	2015–2020	−13.9 (−19.1, −8.3)	0.001					
30-	2006–2017	−0.8 (−1.8, 3.6)	0.501	−2.6 (−6.3, 1.2)	2006–2018	−2.2 (−4.2, −0.1)	0.040	−4.9 (−9.4, −0.2)
	2017–2020	−14.4 (−28.5, 2.5)	0.083		2018–2020	−19.6 (−44.2, 15.8)	0.211	
35-	2006–2013	−1.5 (−6.4, 1.4)	0.270	−4.1 (−5.9, −2.3)	2006–2020	−6.0 (−7.6, −4.5)	<0.001	−6.0 (−7.6, −4.5)
	2013–2020	−6.6 (−9.5, −3.6)	0.001					
40-	2006–2008	−11.8 (−29.6, 10.6)	0.231	−5.4 (−8.6, 2.0)	2006–2008	−12.4 (−31.9, 12.7)	0.254	−6.3 (−10.1, −2.4)
	2008–2014	3.5 (−1.3, 8.6)	0.129		2008–2013	4.9 (−3.1, 13.6)	0.197	
	2014–2020	−11.4 (−15.0, −7.8)	<0.001		2013–2020	−11.9 (−15.1, −8.5)	<0.001	
45-	2006–2012	4.6 (0.8, 8.6)	0.023	−0.7 (−2.4, 1.0)	2006–2011	4.4 (−4.0, 13.7)	0.278	−1.1 (−4.2, 2.1)
	2012–2020	−4.6 (−6.5, −2.6)	<0.001		2011–2020	−4.1 (−6.9, −1.2)	0.011	
50-	2006–2011	−1.1 (−9.2, 7.6)	0.762	−1.4 (−5.5, 3.0)	2006–2012	−2.0 (−8.7, 5.1)	0.519	−1.6 (−6.2, 3.2)
	2011–2017	8.8 (1.4, 16.8)	0.025		2012–2017	9.9 (−0.9, 22.0)	0.069	
	2017–2020	−19.4 (−29.8, −7.4)	0.008		2017–2020	−17.6 (−29.2, −4.1)	0.019	
55-	2006–2013	3.4 (0.3, 6.7)	0.036	1.0 (−2.2, 4.3)	2006–2020	−1.8 (−3.0, −0.6)	0.007	−1.8 (−3.0, −0.6)
	2013–2017	−6.7 (−15.9, 3.6)	0.161					
	2017–2020	6.1 (−3.1, 16.2)	0.168					
60-	2006–2018	4.5 (2.8, 6.1)	<0.001	0.6 (−2.4, 3.7)	2006–2018	3.0 (1.4, 4.5)	0.001	−0.5 (−3.4, 2.5)
	2018–2020	−19.6 (−35.5, 0.1)	0.051		2018–2020	−18.9 (−35.1, 1.2)	0.061	
65-	2006–2017	5.6 (3.7, 7.4)	<0.001	2.4 (0.2, 4.6)	2006–2017	4.3 (1.9, 6.7)	0.002	0.7 (−2.2, 3.6)
	2017–2020	−8.5 (−16.5, 0.4)	0.058		2017–2020	−11.6 (−22.3, 0.6)	0.060	
70-	2006–2020	1.7 (0.4, 3.0)	0.014	1.7 (0.4, 3.0)	2006–2020	0.3 (−1.1, 1.7)	0.625	0.3 (−1.1, 1.7)
75-	2006–2020	0.8 (−0.4, 2.0)	0.187	0.8 (−0.4, 2.0)	2006–2020	−0.7 (−1.8, 0.4)	0.178	−0.7 (−1.8, 0.4)

**Table A2:** The temporal trends in age-specific mortality rates for each age group in rural regions using Joinpoint regression analysis.

	Male				Female			
Age	Range	APC (%, 95% CI)	*p*	AAPC (%, 95% CI)	Range	APC (%, 95% CI)	*p*	AAPC (%, 95% CI)
5-	2006–2012	3.1 (−1.0, 7.3)	0.124	−5.2 (−7.3, −3.0)	2006–2012	3.7 (−2.3, 10.0)	0.201	−3.4 (−6.3, −0.4)
	2012–2020	−10.9 (−13.8, −8.0)	<0.001		2012–2020	−8.4 (−12.1, −4.5)	0.001	
10-	2006–2010	−8.4 (−15.3, −0.9)	0.034	−2.7 (−6.6, 1.4)	2006–2010	−3.0 (−12.9, 8.0)	0.523	1.9 (−5.0, 9.3)
	2010–2014	15.0 (0.3, 32.0)	0.047		2010–2013	22.4 (−14.1, 74.4)	0.220	
	2014–2020	−9.4 (−13.7, −5.0)	0.002		2013–2020	−3.1 (−7.1, 1.1)	0.126	
15-	2006–2011	−2.9 (−5.5, −0.2)	0.038	−4.4 (−7.0, −1.7)	2006–2020	−2.7 (−4.0, −1.4)	0.001	−2.7 (−4.0, −1.4)
	2011–2014	7.2 (−6.4, 22.8)	0.267					
	2014–2020	−10.8 (−13.4, −8.0)	<0.001					
20-	2006–2012	5.4 (1.8, 9.1)	0.009	−2.8 (−6.5, 1.2)	2006–2008	27.4 (−26.0, 119.1)	0.344	−4.4 (−11.2, −3.0)
	2012–2017	−15.2 (−21.2, −8.7)	0.001		2008–2020	−8.8 (−12.2, −5.3)	<0.001	
	2017–2020	4.0 (−12.5, 23.6)	0.606					
25-	2006–2014	9.3 (5.6, 13.2)	<0.001	0.3 (−2.4, 3.0)	2006–2014	4.2 (0.9, 7.6)	0.017	−2.7 (−5.2, 0.0)
	2014–2020	−10.7 (−15.4, −5.7)	0.001		2014–2020	−11.1 (−16.1, −5.8)	0.001	
30-	2006–2009	−7.1 (−28.4, 20.4)	0.522	−0.9 (−7.7, 6.3)	2006–2015	0.7 (−2.8, 4.4)	0.652	−3.0 (−6.7, 0.8)
	2009–2014	12.6 (−2.8, 30.6)	0.099		2015–2020	−9.5 (−18.6, 0.7)	0.063	
	2014–2020	−8.1 (−15.6, 0.1)	0.053					
35-	2006–2020	−3.7 (−4.7, −2.6)	<0.001	−3.7 (−4.7, −2.6)	2006–2020	−6.5 (−7.4, −5.6)	<0.001	−6.5 (−7.4, −5.6)
40-	2006–2012	−0.3 (−5.1, 4.9)	0.906	−5.4 (−8.2, −2.6)	2006–2020	−7.4 (−9.4, −5.3)	<0.001	−7.4 (−9.4, −5.3)
	2012–2020	−9.1 (−13.2, −4.8)	0.001					
45-	2006–2012	9.1 (6.9, 11.3)	<0.001	−0.6 (−1.6, 0.4)	2006–2011	6.6 (−1.7, 15.5)	0.110	−2.2 (−5.2, −0.9)
	2012–2020	−7.3 (−8.5, −6.1)	<0.001		2011–2020	−6.8 (−9.8, −3.7)	0.001	
50-	2006–2018	1.7 (−0.4, 3.9)	0.109	−1.7 (−6.8, 3.8)	2006–2012	−5.7 (−12.0, 1.0)	0.084	−3.8 (−9.0, 1.7)
	2018–2020	−19.5 (−46.5, 20.9)	0.262		2012–2017	7.5 (−4.8, 21.4)	0.202	
					2017–2020	−16.7 (−31.3, 0.9)	0.059	
55-	2006–2008	15.6 (−23.0, 73.5)	0.446	−1.7 (−6.9, 3.8)	2006–2020	−5.0 (−7.1, −2.7)	<0.001	−5.0 (−7.1, −2.7)
	2008–2020	−4.3 (−6.6, −1.9)	0.003					
60-	2006–2009	8.9 (−7.4, 28.0)	0.269	0.6 (−2.7, 4.0)	2006–2008	19.6 (−12.9, 64.2)	0.238	−0.1 (−4.2, 4.2)
	2009–2020	−1.6 (−3.5, 0.4)	0.109		2008–2020	−3.0 (−4.7, −1.3)	0.003	
65-	2006–2017	2.5 (1.2, 3.8)	0.002	0.7 (−1.1, 2.4)	2006–2017	0.8 (−0.1, 1.8)	0.079	−0.9 (−2.2, 0.4)
	2017–2020	−5.9 (−13.1, 2.1)	0.127		2017–2020	−7.0 (−12.5, −1.1)	0.025	
70-	2006–2020	−0.1 (−0.7, 0.5)	0.712	−0.1 (−0.7, 0.5)	2006–2008	5.1 (−4.0, 15.1)	0.247	−0.1 (−1.3, 1.1)
					2008–2020	−1.0 (−1.5, −0.4)	0.002	
75-	2006–2009	6.9 (0.1, 14.1)	0.048	1.4 (−0.6, 3.4)	2006–2009	3.0 (−1.7, 7.8)	0.178	0.3 (−1.1, 1.7)
	2009–2018	−2.9 (−4.2, −1.5)	0.002		2009–2018	−2.7 (−3.7, −1.7)	<0.001	
	2018–2020	13.6 (1.1, 27.5)	0.036		2018–2020	10.5 (1.4, 20.5)	0.029	

**Table A3:** Age–period–cohort analysis of injury and poisoning mortality rates for men and women in urban China, 2006–2020.

	**Male**						**Female**					
	**Coef**	**Std.Err**	**z**	***p***	**95% CI**		**Coef**	**Std.Err**	**z**	***p***	**95% CI**	
					**Lower**	**Upper**					**Lower**	**Upper**
age												
5–9	1.427	0.116	12.270	0.000	1.199	1.655	1.443	0.146	9.900	0.000	1.157	1.728
10–14	0.686	0.093	7.390	0.000	0.504	0.868	0.730	0.116	6.280	0.000	0.502	0.958
15–19	0.043	0.084	0.510	0.612	−0.122	0.207	0.092	0.105	0.880	0.379	−0.113	0.298
20–24	0.202	0.086	2.360	0.018	0.034	0.370	0.226	0.108	2.100	0.036	0.015	0.437
25–29	−0.298	0.093	−3.210	0.001	−0.479	−0.116	−0.270	0.116	−2.320	0.020	−0.497	−0.042
30–34	−0.817	0.100	−8.170	0.000	−1.013	−0.621	−0.808	0.125	−6.450	0.000	−1.053	−0.562
35–39	−0.616	0.105	−5.860	0.000	−0.822	−0.410	−0.657	0.132	−4.990	0.000	−0.915	−0.399
40–44	−0.810	0.107	−7.580	0.000	−1.020	−0.601	−0.847	0.134	−6.320	0.000	−1.109	−0.584
45–49	−0.994	0.105	−9.460	0.000	−1.199	−0.788	−1.009	0.132	−7.670	0.000	−1.267	−0.751
50–54	−0.577	0.100	−5.780	0.000	−0.773	−0.381	−0.633	0.125	−5.060	0.000	−0.879	−0.388
55–59	−0.541	0.093	−5.830	0.000	−0.722	−0.359	−0.563	0.116	−4.850	0.000	−0.791	−0.336
60–64	−0.287	0.086	−3.350	0.001	−0.456	−0.119	−0.293	0.108	−2.720	0.006	−0.504	−0.082
65–69	0.596	0.084	7.110	0.000	0.432	0.761	0.577	0.105	5.500	0.000	0.371	0.783
70–74	0.825	0.093	8.870	0.000	0.642	1.007	0.835	0.116	7.170	0.000	0.607	1.063
75–79	1.160	0.116	10.010	0.000	0.933	1.387	1.177	0.145	8.100	0.000	0.892	1.461
period												
2006–2010	−0.109	0.027	−3.980	0.000	−0.163	−0.056	−0.040	0.034	−1.180	0.240	−0.108	0.027
2011–2015	−0.464	0.026	−17.610	0.000	−0.515	−0.412	−0.449	0.033	−13.620	0.000	−0.514	−0.384
2016–2020	0.573	0.028	20.710	0.000	0.519	0.627	0.490	0.035	14.120	0.000	0.422	0.558
cohort												
1929–1933	−1.590	0.155	−10.290	0.000	−1.893	−1.288	−1.630	0.194	−8.420	0.000	−2.009	−1.250
1934–1938	−1.100	0.112	−9.840	0.000	−1.320	−0.881	−1.140	0.140	−8.140	0.000	−1.415	−0.866
1939–1943	−0.428	0.088	−4.840	0.000	−0.602	−0.255	−0.498	0.111	−4.500	0.000	−0.716	−0.281
1944–1948	0.058	0.084	0.700	0.486	−0.106	0.222	0.029	0.105	0.280	0.781	−0.176	0.235
1949–1953	0.286	0.088	3.240	0.001	0.113	0.459	0.317	0.111	2.870	0.004	0.100	0.534
1954–1958	0.637	0.097	6.560	0.000	0.447	0.827	0.625	0.122	5.130	0.000	0.386	0.863
1959–1963	1.146	0.105	10.880	0.000	0.939	1.352	1.190	0.132	9.010	0.000	0.931	1.448
1964–1968	1.079	0.111	9.730	0.000	0.862	1.296	1.106	0.140	7.960	0.000	0.834	1.378
1969–1973	0.837	0.113	7.420	0.000	0.616	1.058	0.862	0.141	6.100	0.000	0.585	1.139
1974–1978	1.067	0.111	9.640	0.000	0.850	1.284	1.109	0.139	8.000	0.000	0.838	1.381
1979–1983	0.920	0.105	8.770	0.000	0.715	1.126	0.937	0.131	7.130	0.000	0.679	1.195
1984–1988	0.401	0.096	4.160	0.000	0.212	0.589	0.415	0.121	3.430	0.001	0.178	0.651
1989–1993	0.138	0.087	1.580	0.115	−0.033	0.309	0.123	0.109	1.120	0.261	−0.091	0.337
1994–1998	−0.111	0.082	−1.350	0.177	−0.272	0.050	−0.118	0.103	−1.150	0.251	−0.320	0.084
1999–2003	−0.643	0.087	−7.400	0.000	−0.814	−0.473	−0.628	0.109	−5.760	0.000	−0.842	−0.415
2004–2008	−1.062	0.110	−9.650	0.000	−1.278	−0.846	−1.078	0.138	−7.820	0.000	−1.349	−0.808
2009–2013	−1.632	0.164	−9.980	0.000	−1.953	−1.311	−1.620	0.205	−7.900	0.000	−2.021	−1.218
con	−18.082	0.022	−822.780	0.000	−18.125	−18.039	−18.068	0.028	−656.130	0.000	−18.122	−18.014
AIC	−1.181						−0.731					
BIC	−49.291						−49.181					

**Table A4:** Age–period–cohort analysis of injury and poisoning mortality rates for men and women in rural China, 2006–2020.

	Male						Female					
	Coef	Std.Err	z	*p*	95% CI		Coef	Std.Err	z	*p*	95% CI	
					Lower	Upper					Lower	Upper
age												
5–9	0.628	0.228	2.760	0.006	0.181	1.074	0.670	0.209	3.210	0.001	0.261	1.079
10–14	0.321	0.182	1.760	0.078	−0.036	0.677	0.369	0.167	2.210	0.027	0.042	0.695
15–19	0.139	0.164	0.85	0.397	−0.183	0.461	0.151	0.150	1.000	0.315	−0.144	0.446
20–24	−0.153	0.168	−0.910	0.361	−0.483	0.176	−0.145	0.154	−0.940	0.348	−0.446	0.157
25–29	−0.228	0.181	−1.260	0.209	−0.584	0.128	−0.214	0.166	−1.280	0.199	−0.539	0.112
30–34	−0.340	0.196	−1.740	0.082	−0.723	0.044	−0.332	0.179	−1.850	0.064	−0.684	0.019
35–39	−0.518	0.206	−2.52	0.012	−0.921	−0.115	−0.525	0.188	−2.790	0.005	−0.894	−0.156
40–44	−0.413	0.209	−1.970	0.049	−0.823	−0.003	−0.413	0.192	−2.160	0.031	−0.789	−0.038
45–49	−0.391	0.206	−1.900	0.057	−0.794	0.012	−0.397	0.188	−2.110	0.035	−0.766	−0.028
50–54	−0.330	0.196	−1.690	0.092	−0.713	0.054	−0.392	0.179	−2.190	0.029	−0.744	−0.041
55–59	−0.316	0.182	−1.740	0.082	−0.671	0.040	−0.313	0.166	−1.880	0.060	−0.639	0.013
60–64	−0.047	0.168	−0.280	0.779	−0.377	0.282	−0.019	0.154	−0.120	0.903	−0.321	0.283
65–69	0.282	0.164	1.720	0.086	−0.040	0.604	0.237	0.150	1.570	0.116	−0.058	0.531
70–74	0.592	0.182	3.250	0.001	0.235	0.948	0.555	0.167	3.330	0.001	0.228	0.882
75–79	0.774	0.227	3.410	0.001	0.330	1.219	0.768	0.208	3.700	0.000	0.361	1.176
period												
2006–2010	−0.175	0.054	−3.240	0.001	−0.280	−0.069	−0.101	0.049	−2.060	0.040	−0.198	−0.005
2011–2015	−0.283	0.052	−5.480	0.000	−0.384	−0.181	−0.308	0.047	−6.510	0.000	−0.400	−0.215
2016–2020	0.457	0.054	8.440	0.000	0.351	0.563	0.409	0.050	8.240	0.000	0.312	0.506
cohort												
1929–1933	−0.680	0.303	−2.250	0.025	−1.273	−0.087	−0.735	0.277	−2.650	0.008	−1.278	−0.191
1934–1938	−0.666	0.219	−3.040	0.002	−1.095	−0.237	−0.697	0.201	−3.470	0.001	−1.090	−0.303
1939–1943	−0.137	0.173	−0.790	0.430	−0.476	0.203	−0.173	0.159	−1.090	0.275	−0.484	0.138
1944–1948	0.136	0.164	0.830	0.408	−0.186	0.457	0.139	0.150	0.930	0.354	−0.155	0.433
1949–1953	0.297	0.173	1.720	0.086	−0.042	0.636	0.258	0.159	1.620	0.104	−0.053	0.568
1954–1958	0.332	0.190	1.740	0.081	−0.041	0.705	0.297	0.174	1.700	0.088	−0.044	0.638
1959–1963	0.651	0.206	3.160	0.002	0.247	1.056	0.689	0.189	3.640	0.000	0.318	1.059
1964–1968	0.633	0.217	2.910	0.004	0.207	1.058	0.680	0.199	3.420	0.001	0.290	1.070
1969–1973	0.316	0.221	1.430	0.152	−0.116	0.749	0.331	0.202	1.640	0.101	−0.065	0.728
1974–1978	0.457	0.217	2.110	0.035	0.032	0.882	0.474	0.199	2.390	0.017	0.085	0.864
1979–1983	0.589	0.205	2.870	0.004	0.187	0.992	0.638	0.188	3.390	0.001	0.269	1.006
1984–1988	0.168	0.189	0.890	0.373	−0.202	0.538	0.192	0.173	1.110	0.266	−0.146	0.531
1989–1993	0.012	0.171	0.070	0.945	−0.323	0.347	0.019	0.157	0.120	0.905	−0.288	0.326
1994–1998	−0.037	0.161	−0.23	0.820	−0.352	0.279	−0.015	0.148	−0.100	0.920	−0.304	0.274
1999–2003	−0.358	0.170	−2.100	0.036	−0.692	−0.024	−0.349	0.156	−2.240	0.025	−0.655	−0.043
2004–2008	−0.580	0.216	−2.690	0.007	−1.002	−0.157	−0.617	0.198	−3.120	0.002	−1.004	−0.230
2009–2013	−1.135	0.320	−3.540	0.000	−1.762	−0.507	−1.132	0.293	−3.860	0.000	−1.707	−0.557
con	−18.843	0.043	−437.820	0.000	−18.927	−18.759	−18.803	0.039	−476.970	0.000	−18.880	−18.726
AIC	0.162						−0.013					
BIC	−48.739						−48.859					
